# How fibrosis influences imaging and surgical decisions in pancreatic cancer

**DOI:** 10.3389/fphys.2012.00389

**Published:** 2012-10-02

**Authors:** Mert Erkan, Simone Hausmann, Christoph W. Michalski, Anna M. Schlitter, Alexander A. Fingerle, Martin Dobritz, Helmut Friess, Jörg Kleeff

**Affiliations:** ^1^Department of General Surgery, Klinikum rechts der Isar, Technische Universität MünchenMunich, Germany; ^2^Institute of Pathology, Technische Universität MünchenMunich, Germany; ^3^Institute of Radiology, Klinikum rechts der Isar, Technische Universität MünchenMunich, Germany

**Keywords:** microenvironment, stellate cells, angiogenesis, desmoplasia, periostin, cystic pancreatic tumors, molecular diagnostics

## Abstract

Our understanding of pancreatic ductal adenocarcinoma (PDAC) is shifting away from a disease of malignant ductal cells-only, toward a complex system where tumor evolution is a result of interaction of cancer cells with their microenvironment. This change has led to intensification of research focusing on the fibrotic stroma of PDAC. Pancreatic stellate cells (PSCs) are the main fibroblastic cells of the pancreas which are responsible for producing the desmoplasia in chronic pancreatitis (CP) and PDAC. Clinically, the effect of desmoplasia is two-sided; on the negative side it is a hurdle in the diagnosis of PDAC because the fibrosis in cancer resembles that of CP. It is also believed that PSCs and pancreatic fibrosis are partially responsible for the therapy resistance in pancreatic cancer. On the positive side, a fibrotic pancreas is safer to operate on compared to a fatty and soft pancreas which is prone for postoperative pancreatic fistula. In this review the impact of pancreatic fibrosis on diagnosis of pancreatic cancer and surgical decisions are discussed from a clinical point of view.

## Introduction

**Figure 1:**
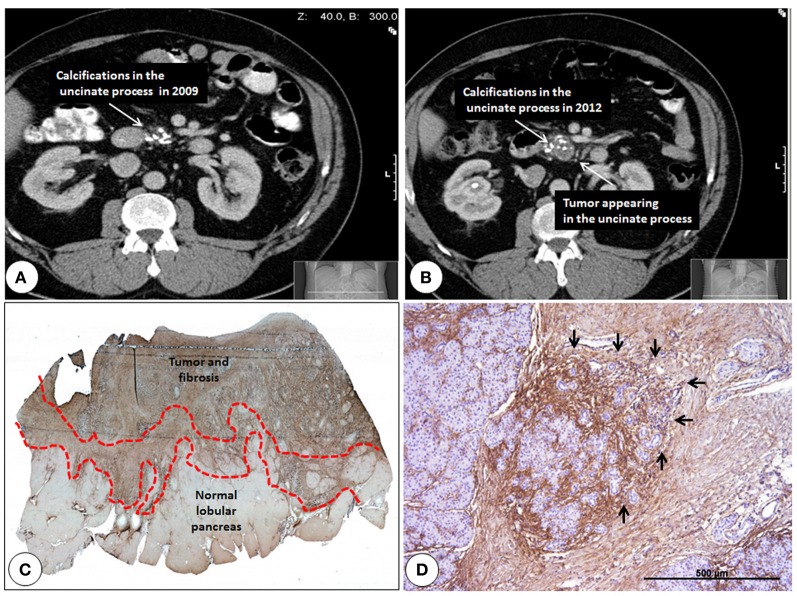
**Pancreatic cancer developing in the background of chronic pancreatitis and immunohistochemistry of the activated stroma between the cancer and the normal pancreas. (A)** Computed tomography of a chronic pancreatitis patient from 2009 showing pancreatic calcifications. **(B)** Computed tomography of the same patients 3 years later showing the increase of tissue around calcifications which is highly suggestive of cancer development. **(C)** Notice the finger-like extension of the activated stroma (marked by dotted lines) between the normal pancreatic lobuli and the cancer. Periostin staining (brown) is made without counterstaining. Original magnification 5x **(D)** strongest periostin expression is found around degenerating acini/atypical flat lesions (black arrows). Original magnification 50x.

Pancreatic fibrosis (desmoplasia) is found both in chronic pancreatitis (CP) and pancreatic ductal adenocarcinoma (PDAC). Pancreatic stellate cells (PSCs) are responsible for producing the desmoplasia in both diseases. Pathophysiologically, desmoplastic replacement of the normal parenchyma leads to the exocrine and endocrine insufficiency of the pancreas. In pancreatic cancer, the fibrotic stroma effect tumorigenesis, angiogenesis, therapy resistance and possibly the metastatic spread of tumor cells (Bachem et al., 2005; Vonlaufen et al., 2008b; Erkan et al., 2009; Xu et al., 2010; Apte and Wilson, 2012). Clinically, the effect of desmoplasia is two-sided; on the negative side it is a hurdle in the diagnosis of PDAC because the fibrosis in cancer resembles that of CP. This becomes even more important considering the fact that CP is a risk factor for PDAC (Figures 1A,B). Here, fibrosis poses a problem for the radiologists, surgeons, and pathologists. As it is radiologically difficult to differentiate between tumor and desmoplasia, identification of tumor borders intraoperatively is also difficult to define. This is getting to be a bigger problem as the frequency of resections after neoadjuvant (radio-) chemotherapy is increasing. In cases where there is a good response to the neoadjuvant therapy, tumor tissue is replaced to a great extent with fibrosis, creating a challenge for the pathologist during the frozen-section analysis. On the positive side, after pancreatic resection, a fibrotic pancreas is safer to anastomose (pancreatic head resection) or to oversew (the pancreatic stump after distal pancreatectomy) compared to a fatty and soft pancreas. Here, pancreatic fibrosis turns out to be an advantage during surgery as a soft pancreas might increase the postoperative fistula rate (Kurohi et al., 2010; Lee et al., 2010). Various technical modifications have been suggested to reduce the postoperative fistula rate in the case of a soft pancreas. Therefore the consistency of the pancreas influences intraoperative decision-making on how to perform the pancreatic anastomosis. Although several aspects of PSC activity and ensuing desmoplasia will be briefly described, this review focuses mainly on PSCs and pancreatic stroma from a clinical point-of view.

## Periacinar fibrosis and chronic pancreatitis like changes around the tumor

Quiescent PSC belong to the stellate cell system consisting of retinoid-storing cells in various organs (Erkan et al., 2012a). In the pancreas, they are located in the periacinar spaces in close proximity to the basal aspect of acinar cells, capillaries, and terminal nerve fibers (Samkharadze et al., 2011; Apte and Wilson, 2012). PSC extend their long cytoplasmic projections along the base of adjacent acinar cells similar to that of pericytes in the mammary gland. With their ability to secrete acetylcholine, it is probable that one of their physiological functions in the healthy pancreas is to form an electromechanical/humoral interface between the nerve endings and the acini, thereby having a role in the exocrine secretion of the pancreas (Phillips et al., 2010). Judging by early activation markers like periostin, one can observe in pancreatic diseases that the initial activation of quiescent PSC and extracellular matrix (ECM) deposition takes place in the periacinar spaces (Erkan et al., 2009, 2012c).This type of CP-like changes surround the tumor like an umbra and infiltrate the normal parenchyma (Figures 1C,D). It is likely that deposition of ECM around the capillaries and nerve endings interfere with the perfusion of the normal parenchyma and loss of normal function (i.e., contraction of PSC and/or secretion of ACh upon stimulation) of the quiescent PSC. Both in humans and genetically engineered mouse models (GEMM), preneoplastic lesions like pancreatic intraepithelial neoplasia (PanIN) and atypical flat lesions are also commonly found in the activated front of the stroma between the normal areas and the tumor (Erkan et al., 2007a, 2012b,c). During chronic inflammation, abundant presence of cytokines, ECM proteins and hypoxia may all play a role in the initiation of carcinogenesis. For example in GEMM, when pancreas specific activation of Kras was induced in adult mice, it did not lead to carcinogenesis (Guerra et al., 2007). However repetitive cerulein injections leading to pancreatitis induced metaplasia, dysplasia and eventually cancer in the epithelial compartment in these adult mice (Guerra et al., 2007). These changes were accompanied by the typical stromal reaction observed in humans.

Similar to the situation in breast cancer, we have previously argued that microenvironment may provide the “second hit” for epithelial cells that possess tumorigenic potential (Sternlicht et al., 1999; Barcellos-Hoff and Ravani, 2000; Radisky and Przybylo, 2008; Erkan et al., 2012c). Alternatively, the abnormal interactions might lead to genomic instability within the epithelial cells and the acquisition of tumorigenic potential (Barcellos-Hoff and Ravani, 2000; Radisky and Przybylo, 2008; Guturu et al., 2009; Masamune et al., 2009; Pandol et al., 2009; Radisky and Radisky, 2010). The proliferating dysplastic and/or malignant cells can then interact with their microenvironment and enhance the abnormal interactions (Erkan et al., 2007a, 2009). The situation is similar in humans; patients with long-standing CP (e.g., hereditary CP) have an over 25-fold increased risk of developing pancreatic cancer compared with the normal population, probably owing to long-standing inflammation (Lowenfels et al., 1993).

## What is the reason for pancreatic fibrosis in pancreatic ductal adenocarcinoma?

**Figure 2:**
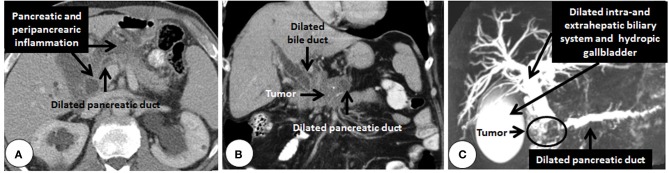
**Cross sectional imaging showing secondary changes due to pancreatic cancer. (A)** Computed tomography of a pancreatic head adenocarcinoma (histologically confirmed after resection) obstructing the pancreatic duct and creating pancreatitis which makes the tumor very difficult to detect. The typical double duct sign created by pancreatic head tumor detected by computed tomography **(B)**. Notice the dilated bile-and pancreatic-ducts failing to merge. The locally advanced tumor is not directly visible in the background of pancreatitis (calcifications appear as white spots in the pancreatic head). **(C)** Reconstruction of T2-weighted imaging (magnetic resonance cholangio-pancreatography) showing the same phenomenon in another patient (fluids appear white in T2-weighted sequences).

**Figure 3:**
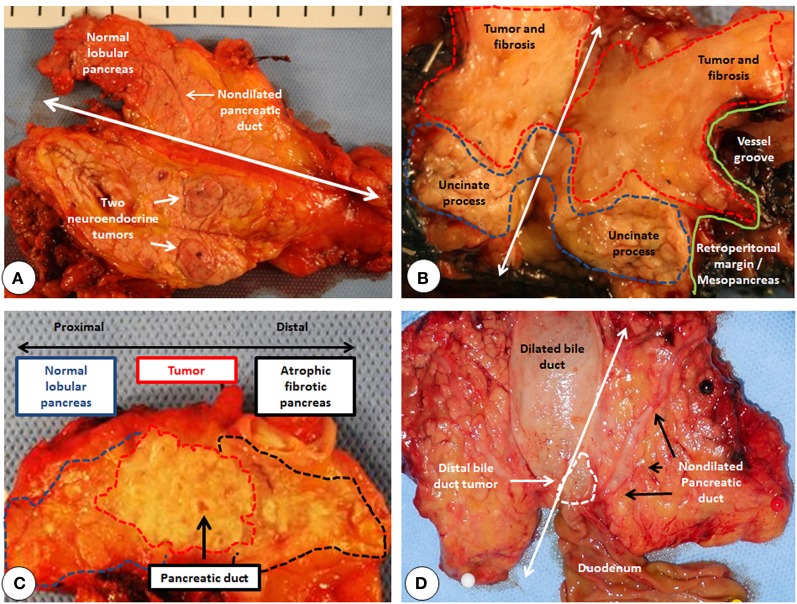
**Panreatic fibrosis is influenced by the localization and the type of the tumor. (A)** The resected pancreatic body is cut open along the white double headed arrow. Notice the lack of fibrosis around the neuroendocrine tumors. **(B)** The resected pancreatic head is cut open along the white double headed arrow. Red dotted line marks the desmoplastic pancreatic head tumor, where it is macroscopically not possible to differentiate between the tumor and the pancreatic fibrosis. Blue dotted line marks the preserved normal uncinate process. Green line marks the touché marked retroperitoneal margin for the microscopic assessment of the R-Status of the resection. **(C)** The resected pancreatic body is cut open longitudinally in the middle. Notice the proximal demarcation of the desmoplastic tumor from the normal lobular pancreas. Distally however, due to the ductal obstruction and repetitive pancreatitis bouts, pancreas is atrophic and fibrotic. **(D)** The resected pancreatic head is cut open along the white double headed arrow. White dotted line marks the small distal bile duct tumor. Notice the lack of fibrosis and dilatation of the pancreatic duct despite massive dilatation of the bile duct.

Histologically, periampullary tumors arise from four different cell types. Compared to distal bile duct-, papillary-, and duodenal-cancers, PDAC elicits a stronger pancreatic fibrosis. Although painless jaundice is the most frequent symptom in all, due to anatomic reasons, distal bile duct cancers for example obstruct the bile duct before obstructing the pancreatic duct. As PDAC originates from the pancreas, in some cases obstruction of the pancreatic duct precedes biliary obstruction. Such patients present sometimes with acute pancreatitis and pose a challenge to the clinician as it is very difficult to find the tumor in the acutely inflamed pancreas (Figure 2A). Most of the pancreatic head tumors (at the time of diagnosis) obstruct both ducts and result in the typical double-duct-sign, that is a dilated bile duct and a dilated pancreatic duct failing to merge due to the mass effect of the tumor (Figures 2B,C). Although the prognosis of PDAC is worst among the four, PDAC has in general the lowest fistula rate after pancreatic head resection due to a simple difference (Bartoli et al., 1991). The pancreas is more fibrotic in PDAC cases compared to the others. This observation is of course unavoidably biased. A distal bile duct tumor (due to its localization) becomes symptomatic (jaundice) in an earlier stage than a PDAC that secondarily obstructs the bile duct. Hence the duration of the disease is different in the two scenarios. But why does even a small PDAC elicit more fibrosis than a distal bile duct cancer does? And why does for example neuroendocrine cancers of the pancreas elicit no fibrosis? (Figure 3A) The answer is two-fold. Pancreatic cancer cells activate the PSC around them, and this activation leads to the fibrosis of the tumor. There is plenty of *in vitro* data supporting this observation. Alternatively, since PDAC originates in the pancreatic ductal system, it obstructs the smaller ductules or even the main pancreatic duct as an early event. PSC distal to the lesion are activated as a consequence of pancreatitis due to ductal obstruction and create pancreatic fibrosis (Tanaka et al., 1988; Panozzo et al., 1995; Kloppel et al., 2004; Erkan et al., 2007a, 2012b,a). The desmoplasia in PDAC is a mixture of both mechanisms. Since most of the PDAC cases are located in the pancreatic head (Figure 3B), the resected tissue after a Whipple's operation contains almost always the part of the tumor-free pancreas distal to the tumor (with the exception of tumors confined to the uncinate process). The pathological analysis of the resection margin shows almost invariably CP like changes. Due to the overlap of both mechanisms in pancreatic head PDAC, it is not possible to dissect how much of the fibrosis distal to the tumor is due to the activation of the PSC directly by cancer cells and how much of it is secondary to ductal obstruction and pancreatitis. To answer this question one should look at the PDAC cases located in the body of the pancreas. In such cases, the proximal part of the tumor-free pancreas is not as fibrotic as the distal part. Proximally, in many cases, one can see a sharp margin between the normal parenchyma and the fibrotic tumor. This margin is more difficult to see distally (Figure 3C). This difference is due to the absence of ductal obstruction and pancreatitis proximal to the tumor and shows the real extent of cancer specific fibrosis. Therefore, distal bile duct tumors which become symptomatic due to jaundice create almost no fibrosis in the normal pancreas increasing the fistula rate after a pancreatic head resection (Figure 3D).

## How does pancreatic fibrosis influence intraoperative decision-making?

Perhaps the most serious complication after pancreatic resections is the pancreatic fistula. It is a major source of postoperative morbidity and is associated with several further complications, such as intra-abdominal abscess, wound infection, sepsis, malabsorption, and haemorrhage (Knaebel et al., 2005). Sepsis and hemorrhage after pancreaticoduodenectomy are associated with a mortality rate of 20–40% (Shrikhande et al., 2005). Pancreatic fistula and its consequences lead to decreased quality of life, loss of work power, development of resistant bacteria, and a financial burden for the health system (Diener et al., 2011). Soft and friable pancreatic parenchyma, makes the anastomosis difficult to perform. A review of 2644 patients in 1991 reported a fistula rate of 5% in CP, 12% in pancreatic cancer, 15% in ampullary cancer, and 33% in bile duct cancer (Bartoli et al., 1991). Other studies have supported the association of soft pancreas with higher leak rates (Sato et al., 1998). As of today there are various attempts to reduce the postoperative pancreatic fistula rate. Such attempts range from the perioperative usage of Octreotide© (somatostatin analog) to modifications of the surgical technique (see below). Regarding the inhibition of pancreatic secretion to reduce pancreatic fistula rate; a recent systematic review and meta-analysis concluded that there is no solid evidence that somatostatin analogs result in a higher closure rate of pancreatic fistula compared with other treatments (Gans et al., 2012).

## Technical modifications to reduce pancreatic fistula rate after pancreatic head resections

**Figure 4:**
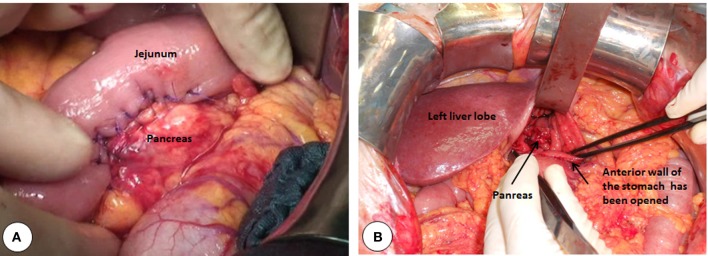
**Intraoperative pictures of various pancreatic anastomosis. (A)** Pancreas is anastomosed to a diverted jejunal loop. **(B)** Pancreas is inserted in the posterior wall of the stomach. The anastomosis is made through a separate incision made on the anterior wall of the stomach.

Pancreatic head resections are mostly performed due to cancer, followed by CP, cysts, trauma, or infiltration of duodenum by other cancers (Lillemoe et al., 1999; Kleeff et al., 2007). After a pancreatic head resection the distal pancreas has to be connected with the alimentary tract. This step is the most critical part of the Whipple's operation. The most commonly performed type of anastomosis is made with a jejunal loop (Figure 4A). Alternatively the posterior wall of the stomach is used to implant/anastomose the distal pancreas (Figure 4B). Although many non-randomized observational clinical studies show the superiority of pancreaticogastrostomy over pancreaticjejunostomy, randomized-controlled trials failed so far to show the superiority of either technique (Yeo et al., 1995b; Bassi et al., 2005b; Duffas et al., 2005; Wente et al., 2007; Fernandez-Cruz et al., 2008). Clinically relevant pancreatic fistula rate (grade B or C in International Study Group on Pancreatic Fistula classification), (Bassi et al., 2005a) after pancreatic head resections is generally less than 15% in reference centers (Yeo et al., 1995a; Bassi et al., 2005b; Winter et al., 2006; Fernandez-Cruz et al., 2008).

There are a number of detailed reviews on the various techniques on how to reduce pancreatic leak rate after pancreatic resections (Shrikhande et al., 2005; Poon and Fan, 2008). Therefore, we will briefly mention some techniques that have failed to decrease the leak rate and focus on three randomized controlled trials (two on pancreatic head resections and one on distal pancreatectomy) where a significant reduction was achieved due to technical modifications. Pancreatic duct occlusion has been abandoned due to high rate of complications (exocrine and endocrine pancreatic insufficiency) and low rate of success (Goldsmith et al., 1971; Di Carlo et al., 1989; Tran et al., 2002).

In order to buttress the anastomosis, well-vascularized structures have been wrapped around the anastomosis. The most commonly used tissues are the omentum and the falciform ligament. Recently, the Japanese Society of Pancreatic Surgery (JSPS) performed a nationwide survey to evaluate whether wrapping using the omentum/falciform ligament can help to prevent postoperative complications after pancreatic head resections (Tani et al., 2012). Analysis of the data from 2597 cases did not show any reduction of fistula by wrapping. In fact, a pancreatic fistula occurred in 623 patients (37.3%) in the non-wrapping group, whereas 393 patients (42.8%) developed fistula in the wrapping group (*P* = 0.006). The incidence of a grade B or C pancreatic fistula was also lower in the non-wrapping group than the wrapping group (16.7% vs. 21.5%; *P* = 0.002). However due to the retrospective and non-randomized structure of this study, it is not possible to conclude that wrapping itself is a risk factor. It is likely that the surgeons anticipating complications due to a risky pancreas took this additional measure (Tani et al., 2012).

Internal duct stenting may help to divert pancreatic secretions from the anastomosis and allow more precise placement of sutures, thus protecting the pancreatic duct from suture injury and reducing the chances of inadvertent pancreatic duct occlusion (Shrikhande et al., 2005). Roder et al. has shown in a prospective but non-randomized study in 85 patients where a stented pancreatic duct decreased the pancreatic leakage rate to 29.3% from 68% and reduced the median hospital stay from 29 to 13 days (Roder et al., 1999). However these results have not been confirmed by randomized studies (Winter et al., 2006).

On the other hand, two randomized controlled prospective clinical trials showed significant reduction of pancreatic leakage rate using an external diverting stent after PJ anastomosis (Poon et al., 2007; Pessaux et al., 2011). In the first study by Poon et al. 120 patients undergoing pancreaticoduodenectomy with end-to-side pancreaticojejunal anastomosis were randomized to have either an external stent inserted across the anastomosis to drain the pancreatic duct (*n* = 60) or no stent (*n* = 60). Duct-to-mucosa anastomosis was performed in all cases. Both groups were comparable in demographic data, underlying pathologies, pancreatic consistency, and duct diameter. In this study stented group had a significantly lower pancreatic fistula rate compared with non-stented group (6.7% vs. 20%, *P* = 0.032). On multivariate analysis, no stenting and pancreatic duct diameter <3 mm were significant risk factors of pancreatic fistula (Poon et al., 2007).

More recently, Pessaux et al. showed similar results on 158 patients in a randomized prospective controlled trial with soft pancreas and a diameter of wirsung <3 mm. (Pessaux et al., 2011). Again, both groups were comparable in terms of demographic data, underlying pathologies, presenting symptoms, presence of comorbid illness, and proportion of patients with preoperative biliary drainage. Also in this study, stented group had a significantly lower overall pancreatic fistula (26% vs. 42%; *P* = 0.034).

In our opinion pancreatic surgeons must have more than one technique for managing the pancreatic stump in their armamentarium. It is nevertheless not possible to eliminate pancreatic fistula completely. Due to the anatomic proximity to major vessels, once they occur, pancreatic fistulas should be controlled as fast as possible to prevent bleeding due to erosion of vessels (i.e., the stump of gastroduodenal artery or portal vein anastomosis after an extensive tumor resection involving the portal vein). As of today, most of the pancreatic fistulas can be managed conservatively or with the help of interventional radiology. However, if a postoperative pancreatic fistula cannot be managed by minimal invasive techniques (i.e., percutaneous drainage), completion pancreatectomy is performed as a salvage operation.

## Technical modifications to reduce pancreatic fistula rate after distal pancreatectomy

Resections of the pancreas reaching to the left of the superior mesenteric vein are defined as distal pancreatectomies. In the 1980 s and 1990 s most distal pancreatectomies were done electively as a result of pancreatitis, other benign diseases, ductal adenocarcinoma, neuroendocrine tumors, and pancreatic pseudocysts. Some remaining small percent were emergency cases after abdominal trauma or miscellaneous pathological diagnoses (Lillemoe et al., 1999). In the last two decades, there is a dramatic surge in performing distal pancreatectomies due to cystic tumors of the pancreas (Kleeff et al., 2007; Beane et al.,^2011^; Diener et al., 2011; Ferrone et al., 2011). Recently the DISPACT trial was designed to assess the effect of stapler versus hand-sewn closure on formation of postoperative pancreatic fistula after distal pancreatectomy (Diener et al., 2011). This multicenter, randomized, controlled trial done in 21 European hospitals recruited 450 patients, of whom 352 patients (177 stapler, 175 hand-sewn closure) were analyzed. Pancreatic fistula rate did not significantly differ between stapler (32%) and hand-sewn closure (49%) (Diener et al., 2011). This study showed however the high fistula rate (grade A–C fistula) of approximately 40% after distal pancreatectomy.

Various attempts have been made to reduce the pancreatic fistula rate after distal pancreatectomy as well. Except of one recent study, none of the randomized controlled trials showed a reduction in pancreatic fistula rate. For example, Frozanpor et al. showed that prophylactic transpapillary pancreatic stent insertion didn't have any effect on clinically significant leak rate following distal pancreatectomy (Frozanpor et al., 2012). Clinically significant pancreatic fistula (Grade B or C) occurred in (22.2%, without stent) and (42.3% with stent *P* = 0.122). In another attempt, two different studies couldn't show any benefit from temporary occlusion of the main pancreatic duct with fibrin glue (Suzuki et al., 1995; Suc et al., 2003).

Recently however, in a single-blinded, parallel-group, randomized controlled trial comparing stapled left pancreatectomy with stapled left pancreatectomy using mesh reinforcement of the staple line with either Seamguard© or Peristrips Dry©, Hamilton et al. could show a significant reduction of ISGPF grade B or C fistula (Hamilton et al., 2012). In this study, clinically significant fistulas were seen in 1.9% of patients undergoing resection with mesh reinforcement and 20% of patients without mesh reinforcement (*P* = 0.0007). According to this study, reinforcing the staple line with some form of mesh buttress material appears to lessen the risk of clinically significant fistula (Hamilton et al., 2012). It is likely that a mesh buttress allows compression and stapling of even a friable pancreas with more safety.

Although an anastomosis between the jejunal loop and the pancreas is a more complex surgical procedure than simple closure of the pancreatic remnant, the fistula rate is significantly higher after distal pancreatectomies compared to pancreatic head resections. The most important factor related to pancreatic fistula is the consistency of the pancreas. As mentioned above, after a pancreatic head resection, the pancreas to be anastomosed is distal to the tumor, hence fibrotic. On the other hand, after a distal pancreatectomy, the pancreas stump to be oversewn is proximal to the tumor hence not fibrotic in most cases.

Due to the low success rate of non-surgical therapies in PDAC, aggressive surgical approaches in reference centers are justified, since resection provides the only chance of cure for some, and the best palliation for most of the patients (Michalski et al., 2008a,b; Kato et al., 2012; Strobel et al., 2012; Winter et al., 2012). Many cases which were not candidates for surgical resection previously are now offered surgical resection within the framework of studies analyzing the effect of neoadjuvant therapy in PDAC. Such cases are especially prone to postoperative complications due to extensive surgery, frequent vascular resections, and due to the negative impact of preoperative radio/chemotherapy on healing, which has already been shown in other tumors (Allendorf et al., 2008; Stumpf et al., 2009; Strobel et al., 2012; Vande Walle et al., 2012). The information on pancreatic stroma after radiotherapy is very limited. Ishikawa et al. were the first ones to show that the risk of pancreatic fistula decreased with preoperative radiotherapy as it decreased the pancreatic secretion (Ishikawa et al., 1991). We have previously shown that PSC are activated *in vitro* after radiotherapy and resected tissues of patients after radiotherapy contain acellular areas of fibrosis (Erkan et al., 2007a). However the detrimental effect of radiotherapy on the healing is mostly due to the thrombosis of the vessels in the target field masking the influence of tissue fibrosis enabling a safer anastomosis. Although rarely performed, patients undergoing extensive surgery are always preoperatively informed about the possibility of performing a total pancreatectomy to prevent a risky anastomosis where vascular resections are concomitantly performed. During the operation, one of the most important factors that the surgeon takes into consideration while performing pancreatic surgery is the consistency of the pancreas. In our and others' experience, a high fat content and a fragile pancreas are more risky to operate than a fibrotic pancreas. In line with this observation Lee et al. have quantitatively analyzed the degrees of pancreatic fatty infiltration and fibrosis preoperatively using magnetic resonance imaging (MRI) (Lee et al., 2010). They detected that patients with a fatty pancreas developed more often postoperative pancreatic fistula. Recently, in a single-center prospective observational study, Ansorge et al. have stratified the pancreas consistency and pancreatic duct diameter in patients undergoing a pancreaticojejunostomy during pancreatic head resection (Ansorge et al., 2012). The morbidity rate attributable to pancreaticojejunostomy was 22% and the clinically relevant fistula rate (grade B or C) was 16%. They could show that both soft pancreas and small pancreatic duct size were risk factors for pancreatic fistula (Ansorge et al., 2012). They concluded that a high risk pancreas (soft and small caliber duct) had a 25-fold increased risk for postoperative fistula compared to low risk pancreas (hard, big caliber duct) (Ansorge et al., 2012). In a former study, Gaujoux et al. have shown that BMI > 25, fatty pancreas and absence of fibrosis in the pancreatic remnant were significant risk factors for pancreatic fistula (Gaujoux et al., 2010). Taken together these data suggest that preoperative radiological or intraoperative clinical evaluation by an experienced surgeon showing lack of fibrosis and fatty pancreas may influence the surgical decision-making such as anastomosis type, performing a total pancreatectomy, stenting the pancreatic duct, or usage of Octreotide© postoperatively to reduce/prevent the pancreatic fistula rate. Whether these measures influence the outcome is a matter of ongoing debate.

According to Okabayashi et al., other factors may influence the rate of fistula after pancreatic head resections. In a retrospective analysis of 50 cases undergoing pancreatoduodenectomy, multivariable analysis identified (in addition to the absence of fibrotic texture of the pancreas), elevated serum amylase levels (more than 1.7-fold of the normal value) on the first postoperative day, and not having early postoperative enteral nutrition as risk factors for developing pancreatic fistula (Okabayashi et al., 2007). It is known that ongoing pancreatitis is a risk factor for healing after pancreatic resection (Erkan et al., 2007b). Therefore, it is likely that elevated serum amylase levels on the first postoperative day are due to some sort of pancreatic inflammation endangering the anastomotic integrity. The authors conclude that early enteral feeding may reduce the rate of pancreatic fistula (Okabayashi et al., 2007). However, failing to initiate enteral nutrition timely may hint (as a surrogate marker) a complicated postoperative phase and not be *per se* a positive factor impacting on the healing of the anastomosis. Due to the low number of cases, retrospective type of analysis, the results of this study needs further validation.

## Antifibrotic therapy in pancreatic cancer: theoretic benefit and risks

Most of the research on PSC has focused on their roles in the diseased pancreas. The amount of information on the role of quiescent PSC in the normal pancreatic physiology is scarce. This is mainly due to spontaneous activation of PSC on plastic during cultivation. Once the PSC are activated it is not easy to revert this myofibroblast-like phenotype into quiescence again. It is unknown how much resemblance exists between the activated PSC *in vitro* to that of physiologic activation *in vivo*. In experimental models, there is a symbiotic relationship between pancreatic cancer cells and PSC (that are already activated on plastic) that results in an overall increase in the growth rate and therapy resistance of the tumor (Erkan et al., 2010). For example, orthotopic cancers induced in nude mice grow faster and have an increased number of regional and distant metastases when pancreatic cancer and stellate cells are injected together (Bachem et al., 2005; Hwang et al., 2008; Vonlaufen et al., 2008a,b; Xu et al., 2010). There is a general belief that PSC are coopted by cancer cells to foster malignancy, thus antifibrotic therapies are suggested to improve the response rate of PDAC (Erkan et al., 2007a; Vonlaufen et al., 2008b; Diop-Frimpong et al., 2011; Apte and Wilson, 2012). One can find dramatic titles in the literature like “Dangerous liaisons: PSCs and pancreatic cancer cells,” “ PSCs and pancreatic cancer cells: an unholy alliance,” “PSCs: partners in crime with pancreatic cancer cells” supporting the premalignant role of PSC (Vonlaufen et al., 2008a,b; Apte and Wilson, 2012).

It is a reality that conventional and targeted therapies which work wonderfully *in vitro* or in animal experiments, fail to show a similar effect in clinical trials (Kessenbrock et al., 2010; Kindler et al., 2010; Conroy et al., 2011). This observation can be explained by various differences between human and mouse pancreatic tumors. For example the pattern of onset of cancer is completely different in GEMM than in humans (focal in humans, global and synchronized in mouse models). We have previously argued that the discrepancy between experimental results and the clinical reality might in part result from the inefficiency of our current experimental setups and animal models in recreating the tumor microenvironment and the fibrotic stroma of PDAC. There is a new trend toward antifibrotic therapies combined with chemotherapy (Erkan et al., 2012b,c). The preliminary encouraging results of such therapies are coming from genetic mouse models for PDAC. In GEMM, antifibrotic therapies (i.e., inhibition of hedgehog signaling or enzymatic digestion of ECM components) applied concomitantly with chemotherapy lead to an increase in the drug penetrance to the tumor and longer survival of the tumor bearing animals (Olive et al., 2009; Jacobetz et al., 2012). The main rationale behind addition of antifibrotic therapy to chemotherapy in PDAC is to make cancer cells which are scattered in a safe haven of fibrosis more accessible for chemotherapeutic agents. As of today data from clinical studies are largely missing. However, as a proof of principle, Von Hoff et al. used in a Phase I/II trial nanoparticle albumin-bound (nab) paclitaxel (to deplete the stoma in PDAC) alone and in combination with gemcitabine and showed that through depletion of stroma, higher concentrations of gemcitabine can be delivered in the tumor (Von Hoff et al., 2011).

Although these arguments are valid, the most important problem in PDAC is late diagnosis. It remains unknown if such antifibrotic therapies combined with chemotherapy could convert locally irresectable tumors to resectable ones. Unfortunately, most of the patients have already distant metastasis at the time of diagnosis and even many of the resectable ones have subclinical metastatic disease. Considering this late time of diagnosis, antifibrotic therapies at this stage could be a double-edged sword. It is not known for sure whether fibrosis acts only as a barrier for chemotherapy or also as a defense against tumor spread. Why should a normal cell support carcinogenesis? As of today, there is no data showing any mutations in PSC originating from PDAC. The innate reaction to form fibrosis is to build a barrier between any noxious stimuli and the body. Fibrosis happens in the form of callosity if one wears a shoe that does not properly fit. It is commonly observed from the feet of the ballerina to the hands of the workers. Fibrotic capsule is found around parasitic cysts (Filippou et al., 2004). In tumor biology, fibrotic capsule formation is a defensive reaction coming from the stroma around the tumor, and tumors with a capsule have better prognosis than infiltrative tumors without a capsule (Lunevicius et al., 2001). In PDAC, collagen deposition has a favorable impact on patient survival (Erkan et al., 2008). At the first glance these arguments may seem to contradict *in vitro* and other experimental (animal) data showing the cooption of PSC to support tumor growth. In fact they do not.

According to the prevailing model of tumor progression, human tumors develop through a succession of genetic and epigenetic changes that confer increasingly malignant characteristics on cells. In 2000, Hanahan and Weinberg distilled properties of cancers into six essential alterations in cell physiology that collectively dictate malignant growth: self-sufficiency in growth signals, insensitivity to growth-inhibitory (antigrowth) signals, evasion of programmed cell death (apoptosis), limitless replicative potential, sustained angiogenesis, and tissue invasion and metastasis (Hanahan and Weinberg, 2000). This review was updated in 2011 and a significantly greater focus on the importance of the tumor microenvironment was added (Hanahan and Weinberg, 2011). Gatenby and Gillies argue that these steps are necessary but not sufficient to produce invasive cancer. In their opinion carcinogenesis requires tumor populations to surmount six distinct microenvironmental proliferation barriers that arise in the adaptive landscapes of normal and premalignant populations growing from epithelial surfaces (Gatenby and Gillies, 2008). These barriers are: apoptosis with loss of basement membrane contact, inadequate growth promotion, senescence, hypoxia, acidosis, and ischemia (Gatenby and Gillies, 2008). Somatic evolution of invasive cancer can then be viewed as a sequence of phenotypical adaptations to these barriers. For example, in PDAC, negative selection of hypoxia resistant clones that down-regulate Bnip3 (a hypoxia inducible pro-apoptotic member of the Bcl-2 family), gain cross-resistance against gemcitabine, and 5-FU (Erkan et al., 2005). Importantly this selection occurs gradually onward from PanIN II, through PanIN III into PDAC (Erkan et al., 2005). In human tissues and GEMM in which PanIN lesions are detectable, a gradual increase in stromal activity and ECM deposition is observed along with progress of the lesion where various genetic mutations accumulate (Erkan et al., 2012b). The fibrosis and hypovascularity are believed to create hypoxia in PDAC (Koong et al., 2000a,b; Erkan et al., 2009). Since the activation of stroma/PSC occurs mostly around preneoplastic lesions like PanIN- or atypical flat-lesions, ideally, the timing of interfering with cancer–stromal interactions and/or elimination of the ECM should not be at the invasive stage, but in earlier stages of carcinogenesis before the adaptation and selection of aggressive clones that can survive in the barren microenvironment of PDAC (Erkan et al., 2012b). As of today the only type of premalignant lesion that can be detected by conventional radiology are the cystic tumors.

## Late diagnosis: the main therapeutic hurdle in the treatment of PDAC

**Figure 5:**
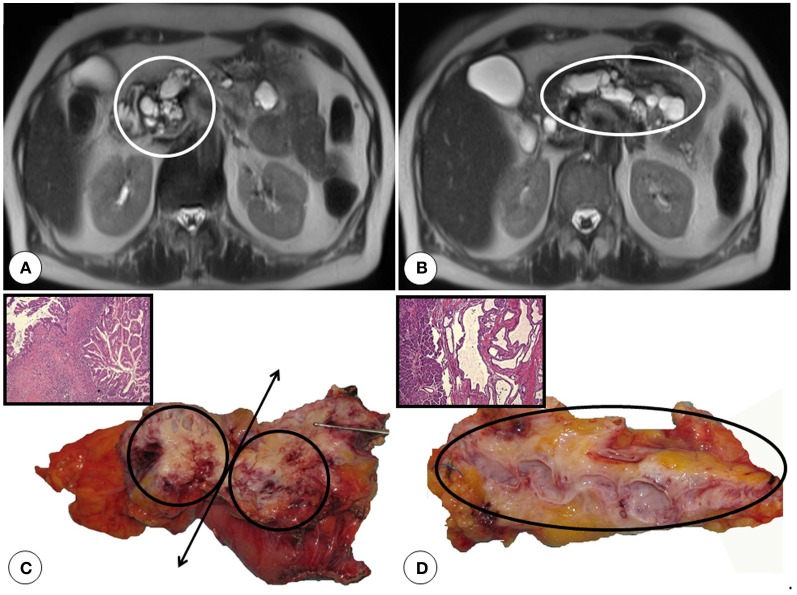
**A case of main duct intraductal papillary mucinous cancer necessitating total pancreatectomy: Fluids appear white in T2-weighted images**. Notice the several cystic lesions in the pancreatic head (white circle, **A**) and the body/tail (white ellipse, **B**). **(C)** The resected pancreatic head is cut open along the black double headed arrow showing the cystic deformities and fibrotic stroma of the invasive cancer. The Hematoxlin and Eosin staining of an area showing invasive cancer with abundant stroma is shown as inset. The circles mark the desmoplastic pancreatic head tumor. **(D)** The resected pancreatic body/tail is cut open longitudinally in the middle. Notice the cystic dilatation of the pancreatic duct and the atrophic and fatty pancreas. Hematoxylin and Eosin staining of a region with high grade intraepithelial neoplasia is shown as inset, original magnification 50x.

**Figure 6:**
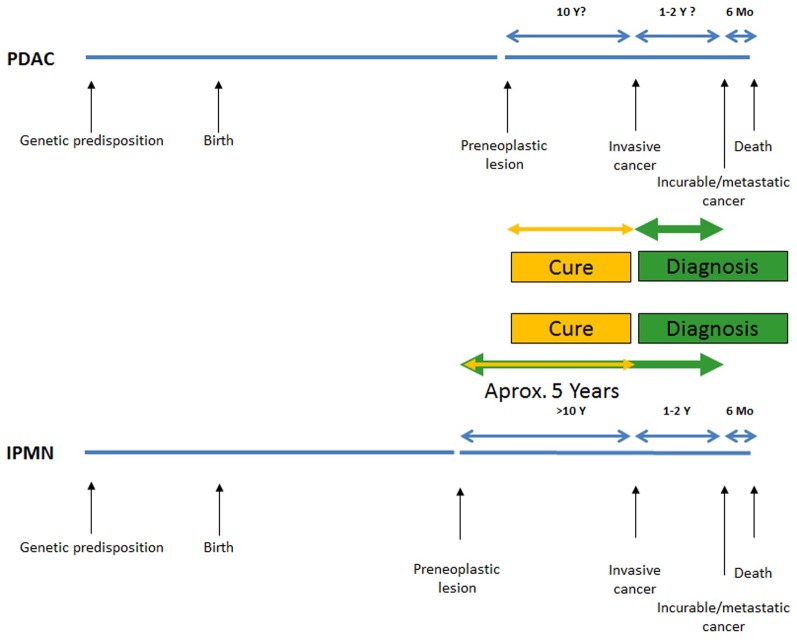
**Schematic comparison of the therapeutic time window between pancreatic ductal adenocarcinoma and the intraductal papillary mucinous neoplasia (Yamao et al., 2000; Salvia et al., 2004; Sohn et al., 2004; Yachida et al., 2010; Matthaei et al., 2011)**.

Due to the insidious course of the disease, retroperitoneal localization of the pancreas (disabling easy access), and lack of specific symptoms and tumor markers suitable for screening (such as PSA in prostate cancer), most pancreatic cancer patients are diagnosed at an advanced stage. Therapeutic time window for a tumor can be described as the period of time from the diagnosis of the earliest removable preneoplastic lesion to the locally advanced- or metastatic-stage of a cancer where cure is no longer possible. For example, with the advent of endoscopic screening, for most of the colorectal cancer cases, the therapeutic time window from the appearance of a removable benign polyp to the development of invasive cancer is on average more than a decade (Fearon and Vogelstein, 1990; Kinzler and Vogelstein, 1996). In contrast, almost all cases of PDAC patients are practically diagnosed at an incurable stage, thereby with a closed therapeutic time window. However in cystic pancreatic tumors, surgery performed at a pre-invasive stage is curative as much as the preventive removal of a premalignant colorectal polyp. Although there are several inherent biological differences between PDAC and cystic malignant pancreatic tumors, once the invasive stage has been reached, the prognosis of intraductal papillary mucinous cancer can be as bad as that of PDAC (Maire et al., 2002; Wada et al., 2005). Therefore, the most important factor impacting on patient survival is the early detection of the lesion at a preinvasive stage. The advantage in the diagnosis of cystic tumors is that they are detectable in a preinvasive stage with conventional radiological methods due to their bigger size (in comparison with PanIN lesions) and the high contrast between the cystic tumor and the normal pancreas (Figure 5). The closed and open therapeutic time windows for cystic pancreatic tumors versus PDAC, respectively is depicted in Figure 6.

## The only conventionally detectable precancerous lesions of the pancreas: cystic tumors

Cystic tumors of the pancreas are being diagnosed with an increasing frequency. It is still unclear if this is due to an increase in the incidence of cystic tumors, or due to an increase in the sensitivity of the diagnostic methods. Laffan et al. reported a frequency 2.6% for unexpected cystic lesions detected in the pancreas during an abdominal tomography performed in a population of adult outpatients imaged for disease unrelated to the pancreas (Laffan et al., 2008). Although there are more than 20 different cystic pancreatic tumor entities, approximately 90% of the lesions are intraductal papillary cystic neoplasms (IPMN), mucinous cystic neoplasms (MCN), solid-pseuodpapillary neoplasms (SPN) or serous cystic neoplasms (SCN) (Fernandez-del Castillo and Warshaw, 2001). SCN have almost invariably a benign course and rarely need surgical therapy (Kosmahl et al., 2004; Matsumoto et al., 2005). On the other hand IPMN, MCN, and SPN have a significant risk or malignant transformation, and surgery is indicated in cases where other criteria are also suggesting malignancy (Kosmahl et al., 2004; Reddy et al., 2004; Salvia et al., 2004; Sohn et al., 2004; Matsumoto et al., 2005; Tang et al., 2005; Tanaka et al., 2006). These cystic tumors of the pancreas are in fact premalignant lesions which are curable if resected in the preinvasive phase. However due to the complexity of pancreatic surgery with a considerable morbidity and mortality, reliable criteria for malignant transformation should be defined. In 2006 Tanaka summarized the consensus guidelines (Sendai criteria) for the management of IPMN and MCN (Tanaka et al., 2006). Depending on the cellular type; IPMN can be gastric type (best survival), oncocytic type, intestinal type, or pancreatobiliary type (worst survival) (Furukawa et al., 2011). Depending on their localization they can be main duct type, branch duct type, or mixed type (Tanaka et al., 2006). Six years later, these criteria have been revised in the light of accumulating data (Tanaka et al., 2012). In this revision, the criteion for characterizing main duct-IPMN has been lowered to main pancreatic duct dilation of >5 mm. “High-risk stigmata” and “worrisome features” have been defined to stratify the risk of malignancy in branch duct-IPMN and consider resection or increased frequency of surveillance. Resection is still recommended in all surgically fit patients with main duct-IPMN or MCN. The indications for resection of branch duct-IPMN became more conservative. Branch duct-IPMNs of >3 cm without “high-risk stigmata” can be observed without immediate resection (Tanaka et al., 2012).

The situation for PDAC in comparison is much worse. As of today perhaps the only lesion that can be described as *in situ* PDAC is PanIN III. However it is almost impossible (excluding some very rare cases of familial PDAC operated on prophylactically) to find PanIN III without PDAC, making the progress from premalignant lesions to overt cancer very difficult to follow. The major problems in detecting early lesions in PDAC are; the small size of the premalignant lesions (micrometer to millimeter) and the similarity of the fibrotic stroma seen both in CP and PDAC masking the tumor in the fibrotic stroma.

## The limits of conventional radiology in detecting cystic and solid tumors of the pancreas

**Figure 7:**
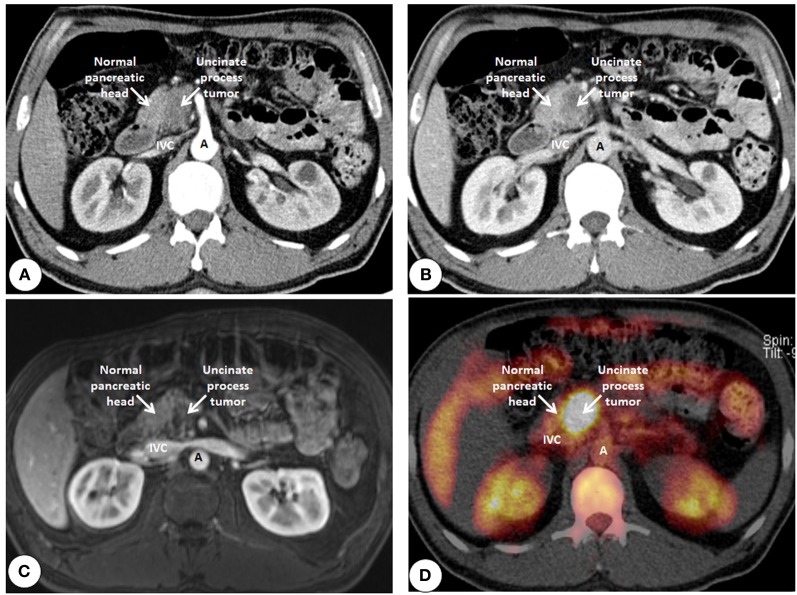
**Cross sectional imaging of an uncinate process ductal adenocarcinoma using tools of conventional radiology. (A)** Computed tomography using intravenous contrast enhancement (arterial phase) shows the delayed contrast uptake of the fibrotic tumor (darker) compared to the normal pancreas (lighter). **(B)** Data acquisition during the venous phase shows an increase of contrast difference due to the reduced perfusion of the fibrotic tumor compared to that of the normal pancreas. **(C)** Contrast enhanced imaging using magnetic resonance imaging showing the tumor also darker than the normal pancreas due to the delay in contrast uptake. **(D)** Positron emission tomography using radiolabeled glucose (Fluorodeoxyglucose^18^) shows the increased tracer uptake in the tumor compared to that of the normal pancreas. IVC, Inferior vena cava; A, Aorta.

As of today, the diagnosis and staging of PDAC is mostly done using computed tomography (CT) or MRI. Although endosonography is a valuable tool in experienced hands, it is also very much user-dependent (Shankar and Russell, 2001; Canto, 2007; Rozen et al., 2009; Canto et al., 2012). Radiologic detection of a tumor is easy when the tumor looks different than the rest of the organ it originates from. The exact principles of how CT and MRI work are beyond the scope of this article. Nevertheless contrast enhancement of the tissues to make tumor and non-tumor appear different is a basic concept for both. Contrast agents are given intravenously and orally to make the vessels and the luminal parts of the gastrointestinal system visible, respectively. In fact, contrast uptake of a tumor is a function of its vascularity, perfusion, and the consistency of its tissue (i.e., fatty, fibrotic). PDAC is a hypovascular tumor and the interactions of PSC and pancreatic cancer cells are believed to be one of the reasons of this hypovascularity *in vivo* (Erkan et al., 2009). Moreover due to increased interstitial pressure within a stiff stroma and frequently encountered pathological vessels, the perfusion of the tumor is further hindered (Perez-Mancera et al., 2012). This hypoperfusion is seen radiologically as delayed contrast uptake in comparison to normal pancreas (Erkan et al., 2012b) (Figures 7A,B). However this hypovascularity is always a relative phenomenon since the amount of fat and fibrosis in the non-tumorous pancreas varies between the patients increasing or decreasing the contrast between the tumor and the “normal” pancreas. In some cases there is no difference between the tumor and the rest of the pancreas despite high suspicion of a tumor (i.e., due to abrupt obstruction of the biliary/pancreatic ductal system). When compared with a good quality contrast enhanced CT, the additional information acquired by MRI is higher in cystic tumors than in PDAC (Figures 5A,B, 7C). As of today the detection limit for both methods is within the range of several millimeters (Holzapfel et al., 2011; Canto et al., 2012).

Since it is sometimes very difficult to differentiate between the tumor and CP where CT and MRI remains equivocal, one can use metabolic imaging. Positron emission tomography (PET) is a non-invasive imaging technique that can assess functional and metabolic activity of normal and diseased tissues. Depending on the tracer used, it makes the evaluation of various cellular functions possible, from glucose metabolism to synthetic capacity of the cell to the resulting tissue hypoxia to cellular division (Juweid and Cheson, 2006; Herrmann et al., 2012). In 1924, the German biochemist Otto Warburg observed that, unlike normal cells, which use oxidative phosphorylation for energy production, cancer cells rely heavily on the less efficient glycolysis to produce ATP. However, by favoring glycolysis over oxidative phosphorylation, malignant cells can spare their pyruvate to make the carbon skeletons necessary for the new nucleic acid, and membrane synthesis required for cellular growth (Kelloff et al., 2005). Another advantage of glycolysis becomes obvious in the oxygen-poor conditions that exist in several solid tumors, which render oxidative phosphorylation less efficient (Jaeschke et al., 2004). This metabolic dichotomy between the normal and cancerous cells allows PET to differentiate between the two. Currently, the most commonly used tracer is the glucose analog fluorine-18 (^18^F) fluorodeoxyglucose (FDG) (Figure 7D). Despite a high sensitivity, FDG has been reported to accumulate also in inflammatory lesions, thereby reducing the specificity of FDG-PET (Buck et al., 2001). New, potentially more specific radiopharmaceuticals for clinical PET imaging have been introduced, such as the thymidine analog 3′-deoxy-3′-[^18^F]fluorothymidine (FLT) (Shields et al., 1998). In 41 patients undergoing pancreatic resection (33 malignant, 8 benign), Hermann et al. showed that, FDG PET and FDG PET/CT showed a higher sensitivity but lower specificity than FLT PET. Interestingly, visual analysis of FLT PET led to two false-positive findings by misinterpreting physiological bowel uptake as pathological FLT uptake in the pancreas (Herrmann et al., 2012). Since PSC also divide in the activated stroma, it is likely that the false-positive signal may partially reflect the stromal proliferation (Erkan et al., 2012c).

## Molecular imaging in PDAC to detect early carcinogenesis

Although the standard methods for diagnosing PDAC have improved due to developments in hardware and software of instruments, the detection limit of MRI, CT /PET-CT, and endosonography is still far beyond the threshold which is needed to detect precancerous lesions at an early stage (Holzapfel et al., 2011; Canto et al., 2012; Herrmann et al., 2012). Recently Canto et al. compared the accuracy of endoscopic ultrasonography, MRI, and CT in detecting pancreatic anomalies in 225 high-risk individuals for developing pancreatic cancer. Among the methods analyzed, endosonography had the best accuracy (43%) followed by MRI (33%) and CT (11%) (Canto et al., 2012). This study also showed the superiority of all methods in detecting cystic tumors compared to solid tumors. Since these results are far from being ideal in detecting early cancer, several research groups are focusing their resources into developing biomarkers and radiological probes for the detection of early cancer using molecular imaging. Since molecular imaging methods are only as accurate as the biomarker used, the identification of a tumor specific biomarker is the first limiting step in molecular imaging. Ideally, the specific target should only be expressed by tumor cells and not in the healthy tissue (or vice versa). After having identified the molecular target, molecular probes have to be developed which specifically bind to the target and amplify the signal. Low toxicity and easy labeling are also important features. Other limiting factors for the successful clinical application for molecular imaging are the impaired tumor vasculature which diminishes the delivery of the probe to the target and overcoming of biological barriers such as the endothelium (Mahmood and Weissleder, 2002). Among many others, cathepsin-based imaging (Cruz-Monserrate et al., 2011; Eser et al., 2011), aptamers (Kim et al., 2011; Lassalle et al., 2012), integrin receptor ligands (Ahmed et al., 2002; Bandyopadhyay and Raghavan, 2009; Kimura et al., 2012), high molecular weight probes such as monoclonal antibodies or recombinant proteins coupled to magnetofluorescent nanoparticles (Zaman et al., 2011; Kelly et al., 2008) have shown promising results in detecting premalignant lesions and/or early stage cancer in experimental settings. Imaging using targeted nanoparticles does not only image the tumor, but also gives the possibility to deliver therapeutic agents. This combined application is termed as “theranostics,” a portmanteau of therapeutics and diagnostics (Picard and Bergeron, 2002).

## Stroma-imaging

**Figure 8:**
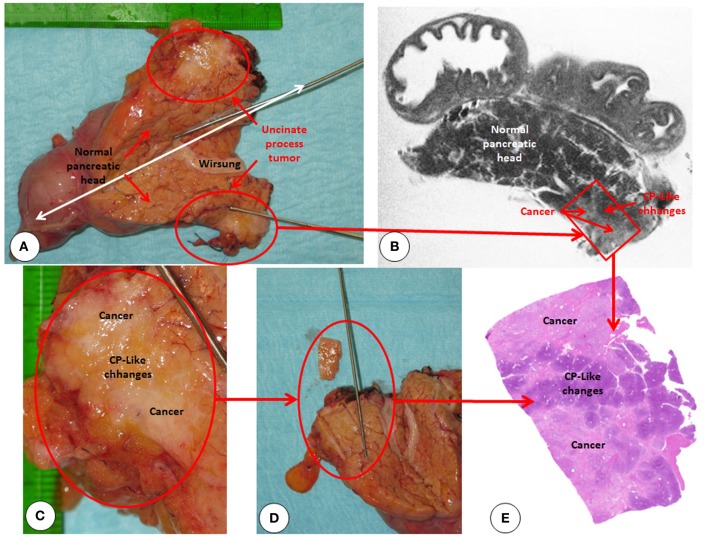
**Correlation of postoperative high resolution T2 weighted magnetic resonance imaging with microscopic findings. (A)** The resected pancreatic head is cut open along the white double headed arrow. Red circle marks the desmoplastic uncinate process tumor. **(B)** High resolution *ex vivo* T2 weighted imaging of the resected specimen using magnetic resonance imaging showing high fidelity of the macroscopic texture. However the resolution is not enough to distinguish early lesions (i.e., PanIN III) with their size of micro- to millimeter. **(C)** Uncinate process in close-up showing the mixture of cancer and chronic pancreatitis-like changes within the stroma. **(D)** Sampling of the tumor for histologic analysis **(E)** Hematoxylin + Eosin staining of the sampled are shows the mixture of tumor and chronic pancreatitis like changes within the tumor stroma.

Since there is a strong stromal reaction around the precursor lesions of PDAC, molecular imaging of the stromal activation might be a promising tool to identify precancerous lesions at very early stages where those can be removed by surgical means. However, molecular imaging of stromal activation is not very well studied so far with differentiating between inflammatory and tumor-activated stroma being the biggest obstacle (Figure 8). Dynamic contrast-enhanced MRI which is used to detect morphologic characteristics of the tumor vasculature is one method which helps to assess the stromal compartment. Farace et al. used for example small molecular and albumin-binding contrast agents and demonstrated that the contrast distribution using dynamic contrast-enhanced MRI was related to the stromal content of the tumor (Farace et al., 2011). Recently Venkatasubramanian have summarized the data on imaging of the ECM (Venkatasubramanian, 2012). The combined use of multiphoton microscopy and second harmonic generation imaging can detect differences in the collagen composition of the ECM (Venkatasubramanian, 2012). In esophageal and breast cancer, it is shown that the stroma of the cancer is “different” than the normal stroma in terms of reorganization of type I collagen fibrils and fibers (Provenzano et al., 2006; Zhuo et al., 2009). However these techniques have not been yet applied to pancreatic cancer.

A promising stroma specific protein which can be used to identify tumor-activated PSC is periostin. This ECM protein is exclusively produced by activated PSC in the pancreas and shows a 42-fold higher expression in PDAC compared to normal pancreas tissue (Erkan et al., 2007a). Furthermore, periostin is a secretory protein that accumulates in the ECM amplifying the amount of target to detect. In humans and in GEMM, highest periostin expression is always found on the activated front of the stroma; between normal pancreas and the tumor. Precancerous lesions like PanIN and atypical flat lesions are commonly found in this zone (Erkan et al., 2012b,c). Therefore, using periostin as biomarker for detecting changes in the stromal compartment has to be assessed in future imaging approaches.

## Conclusion

Our understanding of pancreatic cancer is shifting away from a disease of malignant ductal cells-only, toward a complex system where tumor evolution is a result of interaction of cancer cells with their microenvironment. This change has led to intensification of research focusing on the fibrotic stroma of PDAC. Prior to the identification of PSC and their impact on pancreatic stroma, the presence or absence of pancreatic fibrosis has influenced the decisions of surgeons intraoperatively over many decades. A soft pancreas is a known risk factor for any operation on the pancreas. Appreciation of this phenomenon was mostly a result of trial and error without understanding the mechanisms behind it. As of today, due to better cooperation of clinicians and basic scientist interested in translational research, such clinical problems are better analyzed and solved using tools of molecular biology.

The two main obstacles in the effective treatment of PDAC are late diagnosis and inherent therapy resistance of the tumor. We now understand that both are influenced by the abundant stroma seen in PDAC. The very poor and unchanged prognosis of PDAC patients since years emphasizes the urgent need for early detection methods (Schneider et al., 2005; Jemal et al., 2011). In recent years molecular imaging emerged as a promising tool for detecting precancerous lesions at a very early stage when surgical intervention and chemotherapy is still a therapeutic option. Still, there are some hurdles in the development of molecular imaging methods such as the detection limits that are often too low (Holzapfel et al., 2011; Canto et al., 2012; Herrmann et al., 2012) as well as the toxicity in humans since most of the methods have only been tested in GEMM or xenograft models. Periostin might be a suitable marker for imaging the stroma in PDAC. It is a highly stroma specific protein and its expression precedes that of alpha-smooth muscle actin expression by PSC in humans (Erkan et al., 2007a, 2009).

### Conflict of interest statement

The authors declare that the research was conducted in the absence of any commercial or financial relationships that could be construed as a potential conflict of interest.
